# Methods and Rationale for Using GPS-Derived Objective Technologies to Examine Community Mobility in Older Adults

**DOI:** 10.1093/geroni/igab046.2162

**Published:** 2021-12-17

**Authors:** Breanna Crane, Kyle Moored, Andrea Rosso, Michelle Carlson

**Affiliations:** 1 Johns Hopkins Bloomberg School of Public Health, Baltimore, Maryland, United States; 2 University of Pittsburgh, Pittsburgh, Pennsylvania, United States; 3 Johns Hopkins University, Baltimore, Maryland, United States

## Abstract

Objective measures of community mobility are advantageous for capturing life-space activity. In contrast to subjective, self-reported approaches, GPS-derived objective measures leverage passive, real-time data collection techniques to mitigate recall bias and minimize participant burden. We present methods to quantify community mobility among a sample of 164 community-dwelling older adults (Mean age=77.3±6.5) from a physical therapy intervention aimed at improving walking ability. We characterized community mobility using activity space metrics (e.g., standard deviation ellipse (SDE) area), timing (e.g., time outside home), and shape (e.g., SDE compactness). We will discuss challenges and solutions to generating these metrics as well as their associations with physical and cognitive performance. Time outside of home and SDE area, but not SDE compactness, were correlated with better performance on the 6-Minute Walking Test and Trail-Making Test (Part B) (ρ=.20-.23, p’s<.05). These findings will aid in understanding which community mobility measures are associated with functional capacity.

